# Impact of Oral Targeted Therapy on the Economic Burden of Chronic Lymphocytic Leukemia in Canada

**DOI:** 10.3390/curroncol28010037

**Published:** 2021-01-09

**Authors:** Jean Lachaine, Catherine Beauchemin, Kimberly Guinan, Philippe Thebault, Andrew Aw, Versha Banerji, Isabelle Fleury, Carolyn Owen

**Affiliations:** 1 Faculty of Pharmacy, University of Montreal, Montreal, QC H3T 1J4, Canada; catherine.beauchemin@peripharm.com; 2 PeriPharm Inc., Montreal, QC H2Y 2H4, Canada; kimberly.guinan@peripharm.com (K.G.); philip.thebault@gmail.com (P.T.); 3 Ottawa Hospital, Ottawa, ON K1H 8L6, Canada; aaw@toh.ca; 4 Research Institute in Oncology and Hematology, Cancer Care Manitoba, Winnipeg, MB R3E 0V9, Canada; vbanerji@cancercare.mb.ca; 5 Departments of Internal Medicine and Biochemistry & Medical Genetics, Rady Faculty of Health Sciences, Max Rady College of Medicine, University of Manitoba, Winnipeg, MB R3E 0W2, Canada; 6 Maisonneuve-Rosemont Hospital, Montreal, QC H1T 2M4, Canada; ifleury.hmr@ssss.gouv.qc.ca; 7 Foothills Medical Centre, Calgary, AB T2N 2T9, Canada; Carolyn.Owen@albertahealthservices.ca

**Keywords:** chronic lymphocytic leukemia, oral targeted therapy, economic burden, Markov model

## Abstract

*Background*: Continuous oral targeted therapy (OTT) for chronic lymphocytic leukemia (CLL) represents an effective therapy but also a major economic burden on the healthcare system. This study aimed to estimate future direct costs, along with the prevalence, of CLL in the era of continuous OTT in Canada. *Methods*: The economic burden of OTT was modelled and compared to chemoimmunotherapy (CIT), for CLL treatment. The burden was assessed/projected from 2011 to 2025. For the OTT scenario, CIT was considered the standard of care before 2015, while OTT was considered standard of care for patients with either unmutated immunoglobulin heavy-chain variable (IGHV) or del(17p)/TP53 mutations starting in 2015 and, from 2020 onwards, for all first-line treatments except for patients with mutated IGHV. A Markov model was developed including four health states: watchful-waiting, first-line treatment, relapse and death. Costs of therapy, follow-up/monitoring and adverse events were included. Key clinical parameters were extracted from pivotal clinical trials. *Results*: As incidence rates and rate of survival are increasing, the prevalence of CLL in Canada is projected to increase 1.8-fold, from 8301 patients in 2011 to 14,654 by 2025. Correspondingly, the total annual costs of CLL management are predicted to increase 15.7-fold, from $60.8 million to $957.5 million during that same period. *Conclusions*: Although OTT enhances survival for patients with CLL, it is nonetheless associated with an important economic burden due to the projected vast increase in costs from 2011 to 2025. Changes in clinical strategies, such as implementation of a fixed OTT treatment duration, could help alleviate financial burden.

## 1. Introduction 

Chronic lymphocytic leukemia (CLL) is the most prevalent leukemia in Canada. In 2016, 5900 people were diagnosed with leukemia, of which 44% of cases were CLL [1]. The mean age at diagnosis is approximately 71 years, and, in most patients, CLL is initially managed with a watchful waiting (WW) approach. Treatment is required after a median time of 4.8 years of surveillance and leads to lengthy survival due to the recent availability of highly effective therapies [2]. Several mutations influence treatment and prognosis; including immunoglobulin heavy chain variable region gene (IGHV) mutation status, in which mutated IGHV (mIGHV) responds better to chemoimmunotherapy (CIT) than unmutated IGHV (umIGHV), which has a shorter time to first treatment and an inferior response because of higher resistance to CIT [3]. Patients with a deletion at chromosome 17p targeting the TP53 gene, referred to hereafter as del(17p), also have inferior outcomes, particularly with CIT but less so with novel targeted therapies [3].

Although CIT regimens were the standard first-line treatments for patients with CLL for many years, oral targeted therapy (OTT) has recently emerged as an alternative treatment option. The combination of chlorambucil and obinutuzumab is considered the standard CIT treatment for CLL patients unfit and/or older than 65 years old [4], while the standard CIT regimen for young fit patients is a combination of fludarabine, cyclophosphamide and rituximab (FCR), a regimen that has been shown 3-year progression-free survival (PFS) in 64.0% of patients [5]. The development of OTT has led to substantial advancements in treatment options for CLL, first in the relapse setting in which ibrutinib was first approved in 2015, and later in previously untreated patients. A second OTT, idelalisib, in combination with rituximab, was also approved for the treatment of patients with relapse/refractory CLL but is used infrequently due to adverse events. Oral targeted therapy finds its advantage over CIT not only in superior efficacy, but also through easier administration and reduced hematological toxicity because of its specific targeted mechanism of action. In clinical trials, OTT has shown significant improvement over CIT in PFS, particularly in patients with del(17p) and umIGHV. Thus far, an overall survival advantage of first-line OTT has been seen only in the subgroup of young and fit patients and not in the majority of patients with CLL, who are older or unfit [6,7,8]. This improved efficacy and ease of administration are leading the way for the approval and use of other therapies in the near future.

Despite the demonstrated improvement in PFS with OTT, the high cost associated with these novel therapies raises a concern for payers and patients. Correspondingly, OTT costs on average $100,000 per year and as therapy is continuous and recommended to continue until disease progression or significant toxicity, time on therapy can be lengthy. In addition, because of the lengthy time on therapy and the potential for adverse effects, there is a need for continuous monitoring, which adds an additional cost burden on the public healthcare system. Conversely, standard CIT treatments cost between $20,000 and $45,000 for a fixed treatment duration of 6 months. Adverse events are mostly limited to time on therapy and are generally predictable. The greater expense of treatment with OTT compared with CIT suggests a significant impact on the budget of public and private payers, while co-payments and other expenses (i.e., parking fees, days off work, etc.) may greatly affect patients. To better understand the economic burden of CLL in this new era of OTT, the objective of this study was to predict the future direct costs, as well as the prevalence, of CLL in Canada.

## 2. Methods

### 2.1. Model Structure 

**Figure 1:**
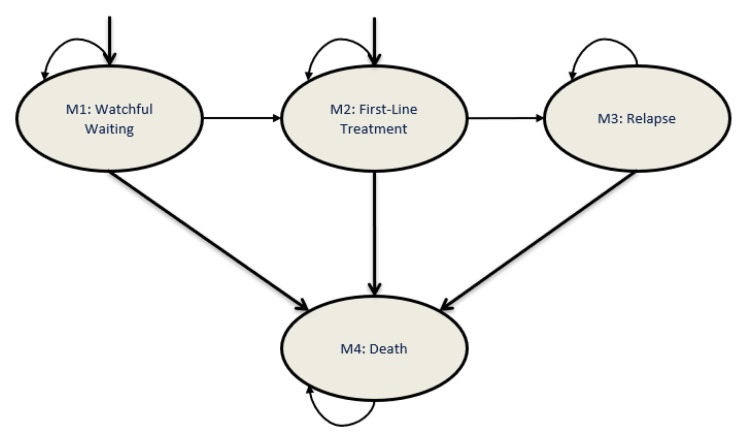
Markov model structure for patients with CLL. The health-state transition model comprises four health states: watchful waiting, first-line treatment, relapse and death. The Markov model developed simulates the course of patients with CLL, who may enter the model from either the watchful waiting or first-line treatment health states. CLL, chronic lymphocytic leukemia.

A Markov model was developed to simulate the CLL patient population under varying treatment strategies in Canada, from 2011 to 2025. Four health states were included in the model: WW, first-line treatment, relapse and death (Figure 1).

### 2.2. Patient Characteristics 

Patients were stratified according to age (<65, 65–70 or >70 years), phase of CLL treatment (WW, first-line or relapse), fitness level and del(17p) or IGHV mutation status. The age at diagnosis was obtained from Statistics Canada by sampling the annual new cases of CLL by age in Canada from 2000 to 2016 [9]. It was estimated that 7% of the total CLL population would be affected by del(17p) mutation [5]. Patients without del(17p) mutation were stratified by age and fitness level. IGHV mutation status was defined for patients aged <65 years old and considered fit. 

In order to estimate the prevalent population living with CLL in 2011, a warm-up period was incorporated into the model. The 2011 population was calculated by adding new incident cases from 2000 to 2010 into the model, stratified using clinical practices from this period. The annual incidence of CLL was calculated using data from Statistics Canada. The calculation of the total number of incident cases was done by applying the annual rate of CLL per patient to the total Canadian population on a yearly basis [10,11]. After this warm-up period, the model had to generate a population reflecting 2011 epidemiologic data. Since the PFS and overall survival (OS) rates extracted from clinical trials are overestimated compared with clinical practice (exclusion of co-morbidities, better stratification, patient ages, etc.), the calculated prevalence was also overestimated. Therefore, the warm-up period served to calibrate the model. The calibration was done using real-world data (prevalence at a specified time, drug utilization, etc.) The main model parameters are presented in Table 1.

### 2.3. Simulated Clinical Pathway 

The model was composed of four health states: WW, first-line treatment, relapse and death. Patients could start in either the WW or first-line treatment states. It was assumed that 85% of patients would enter the model in the WW state, as determined by Chen et al., because the majority of patients with a new diagnosis of CLL do not require immediate treatment [12]. Once patients failed to respond to first-line treatment, they entered the relapse health state. After failure to a second-line treatment, patients entered a sub-health state of relapse, in which their disease progressed but death had still not occurred (palliative state). The possibility of death existed for patients from all health states. Patients within the model were not allowed to revert to previous health states. Cycle length was 28 days over a total time horizon of 15 years. 

The probabilities of health state transitions were estimated based on PFS and OS from pivotal clinical trials and all-cause mortality rates (Table 2). The trials were selected based on the best evidence available, including phase III trials, for the most widely used treatment regimens in clinical practice, referring to product monographs, clinical guidelines as well as key opinion leaders (KOLs). PFS was used to determine the transition from first-line treatment to relapse as well as the progression from relapse (second-line treatment) state. All-cause mortality rates were used to determine the transition to death from WW, first-line treatment and relapse (patients responding to treatment only) health states. Overall survival was used to determine the transition to death of relapse patients who progressed on second-line treatment. The transition rate from WW to first-line treatment was determined through model calibration, using an estimated time to first treatment of 4.8 years [2].

### 2.4. Treatment Patterns

**Figure 2:**
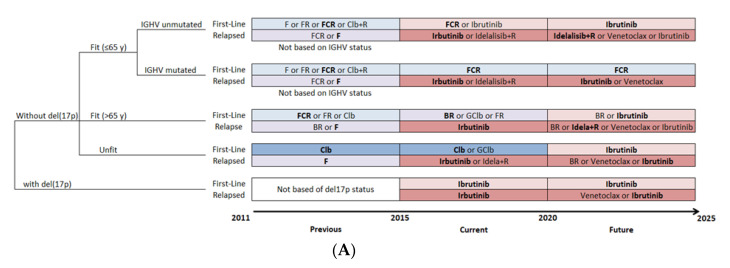

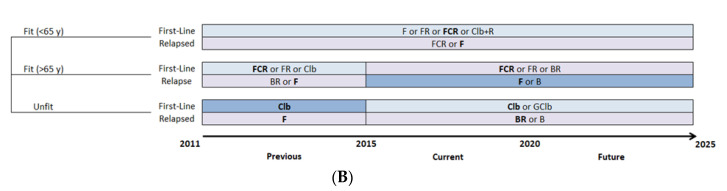
Management strategies for patients with CLL. (**A**) Treatment pattern of OTT scenario, with evolving therapeutic options over time. (**B**) Treatment pattern of CIT scenario, which continues the use of CIT as the standard of care through time. For the base-case analysis, the most widely used therapy was selected when more than one therapy was considered available for patients in the same condition (shown in bold). B, bendamustine; BR, bendamustine and rituximab; CIT, chemoimmunotherapy; Clb, chlorambucil; Clb+R, chlorambucil and rituximab; CLL, chronic lymphocytic leukemia; del(17p), deletion at chromosome 17p targeting the TP53 gene; F, fludarabine; FCR, fludarabine, cyclophosphamide and rituximab; FR, fludarabine and rituximab; GClb, obinutuzumab and chlorambucil; IGHV, immunoglobulin heavy-chain variable; OTT, oral targeted therapy; R, rituximab.

The treatment pattern for CLL was defined for a patient by status of relapse, age, del(17p) mutation, IGHV mutation and year of treatment. The treatment algorithm was simulated from 2011 to 2025, which reflects the evolution of the standard of care and other therapies as well as changes in clinical practice, with the entry of OTT (Figure 2A). A parallel clinical scenario was considered, in which CIT would have remained the standard of care (Figure 2B), to assess the effect of the introduction of OTT as an alternative treatment option. For the CIT scenario, only the age-related fitness level and status of relapse had an effect on the choice of treatment pattern. 

The treatment pattern was based on the Alberta Clinical Guidelines and adapted using the comments of KOLs [28]. The selected treatments were those reimbursed by most Canadian provinces and their entry within the treatment pattern occurred at the time of first reimbursement in a Canadian province. For the OTT scenario, CIT was considered the standard of care before 2015, while OTT was considered standard of care for patients with CLL with either umIGHV or del(17p) mutations starting in 2015 and, from 2020 onwards, for all first-line treatments except for patients with mIGHV.

### 2.5. Cost Data

The model was developed from a public healthcare perspective. For this reason, only direct medical costs were considered, including the costs of drug acquisition, follow-up/monitoring and AEs. Drug costs were calculated based on treatment regimens recommended dosages obtained from product monographs and Cancer Care Ontario (CCO), using a body surface area of 1.89 and an average weight of 76 kg. Unit drug costs were obtained from the Ontario Drug Benefit Formulary and the list of the Association Québécoise des Pharmaciens Propriétaires. For OTT, drug costs were accumulated until treatment discontinuation, due to relapse or other clinical reasons. The probability of discontinuation for each 4-week cycle was estimated at 0.70% and 1.40%, for first-line and relapse patients, respectively [13].

Monitoring cost included the cost of laboratory testing per treatment. The unit costs were obtained from the Schedule of Benefits for Laboratory Services from the Ontario Ministry of Health and Long-Term Care, and the testing frequency was obtained from CCO and validated by KOLs. Administration costs included the cost of chemotherapy infusion as well as professional fee costs, which comprised the hematologist, nurse and pharmacist costs. These costs were calculated based on the Schedule of Benefits of Physician Services in Ontario, the average hourly wage of a nurse and pharmacist obtained from Statistics Canada and the frequency of administration per cycle, as determined by CCO. 

Adverse events were extracted from clinical trials and product monographs for each treatment regimen. Four main AEs were considered for all therapies: neutropenia, thrombocytopenia, anemia and infection. For ibrutinib, arterial fibrillation was included due to specific concerns regarding this treatment. Only grades 3 and 4 AEs were considered in this economic evaluation. The management cost for each type of AE was derived from the Ontario Care Costing Initiative (OCCI), based on the inpatient related data [17].

### 2.6. Model Outcomes

The number of people living with CLL as well as the total costs associated with CLL management in Canada were projected from 2011 to 2025. Additionally, the total costs for first- and second-line treatments and the total cost per patient on an annual basis were calculated for both the OTT and CIT scenarios. All costs were converted to 2019 Canadian dollars. All results are presented in rounded values to simplify comprehension. 

### 2.7. Sensitivity Analyses

The robustness of the results was assessed using one-way sensitivity analyses (OWSA). Within this analysis, model parameters were varied using a range of ±25%. Other model parameters, such as the PFS, OS and probability of discontinuation, were directly varied through calibration in the model; therefore, these values were not included in the sensitivity analyses. 

## 3. Results 

### 3.1. Disease Burden

**Figure 3:**
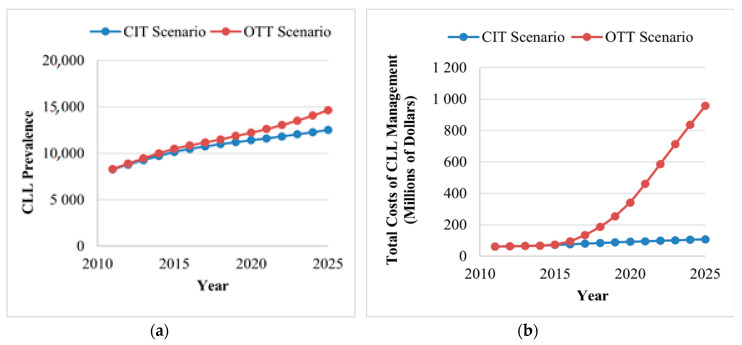
Trends in disease and cost^a^ burden of CLL for the CIT and the OTT scenarios. (**A**) The annual prevalence of CLL (excluding patients in the watchful waiting health state) under the CIT and OTT scenarios. The use of OTT is projected to increase the number of patients living with CLL from 8301 in 2011 to 14,654 by 2025 (1.8-fold increase). (**B**) The total costs of CLL management per year for the CIT and OTT scenarios. The use of OTT is projected to increase the annual costs from $60.8 million in 2011 to $957.5 million in 2015 (15.7-fold increase), which is mainly driven by the increased number of patients with CLL, high drug costs and the increased duration of treatment. CIT, chemoimmunotherapy; CLL, chronic lymphocytic leukemia; OTT, oral targeted therapy. ^a^All costs are shown in 2019 Canadian dollars.

As incidence rates and rate of survival are increasing, the prevalence of CLL in Canada is projected to increase 1.8-fold, from 8301 patients in 2011 to 14,654 by 2025, in the OTT scenario. However, if CIT remains the standard of care, the total number of CLL cases by 2025 is projected to be slightly lower, from 8248 in 2011 to 12,521 in 2025, an estimated 1.5-fold increase (Figure 3A).

### 3.2. Cost Burden

#### 3.2.1. Total Annual Cost of CLL

For the OTT scenario, the total annual cost of CLL management is projected to increase from $60.8 million in 2011 to $957.5 million in 2125, a 15.7-fold increase (Figure 3B). Two major increases in cost were captured in the model. The first increase is observed in 2015 when OTT became available for patients with CLL with either umIGHV or del(17p) mutations. The second surge is observed in 2020, when OTT is projected to become available for all patients receiving first-line treatment, except patients with mIGHV. Compared to the OTT scenario, CIT would also increase but less drastically, reaching $107.6 million (1.76-fold increase) in 2025. When comparing both scenarios, the availability of OTT would result in an additional expenditure of $3.6 billion from 2014 to 2025. The majority of the costs of CLL management are derived from drug acquisition costs. 

#### 3.2.2. Annual Cost per CLL Patient 

The total annual cost of CLL treatment per patient was estimated at $4036 in 2011 and is expected to increase to $43,309 by 2025 (10.7-fold increase) for the OTT scenario. In contrast, costs are predicted to remain quite stable in the CIT scenario, totaling $5202 per patient by 2025 (1.27-fold increase). These costs were calculated by dividing the total cost of CLL per year (Figure 3B) by the annual prevalence of CLL, including patients in the WW health state. 

#### 3.2.3. Cost of First-Line Therapy for CLL

In the OTT scenario, the costs related to first-line treatment are projected to increase from $28.8 million to $490.2 million (17.0-fold increase) from 2011 to 2025, respectively. The costs for the CIT scenario are predicted to remain quite constant, totaling $36.1 million by 2025 (1.3-fold increase). When comparing both scenarios, the introduction of OTT is expected to increase expenses by $1.5 billion from 2011 to 2025. 

#### 3.2.4. Cost of Second-Line Therapy for CLL

In the OTT scenario, the costs related to second-line treatment are expected to increase from $24.6 million to $461.5 million (18.8-fold increase) from 2011 to 2025, respectively. Similar to first-line treatment, the costs related to second-line treatment for the CIT scenario are predicted to remain quite constant, totaling $58.1 million by 2025 (2.4-fold increase). When comparing both scenarios, the introduction of OTT is expected to increase expenses by $2.17 billion from 2011 to 2025.

### 3.3. Sensitivity Analysis

**Figure 4:**
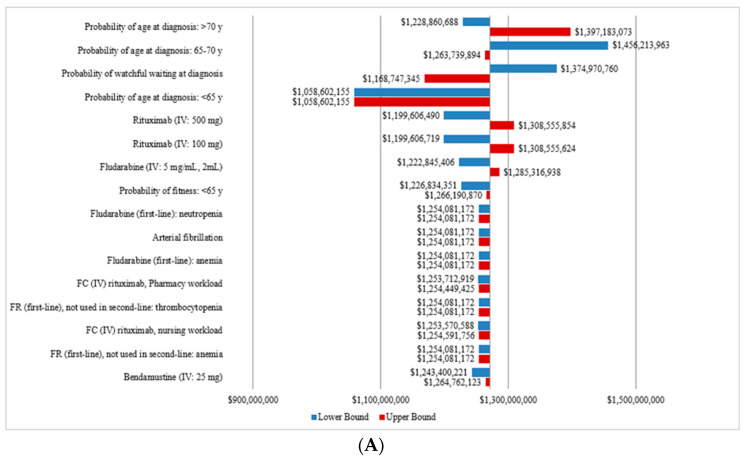

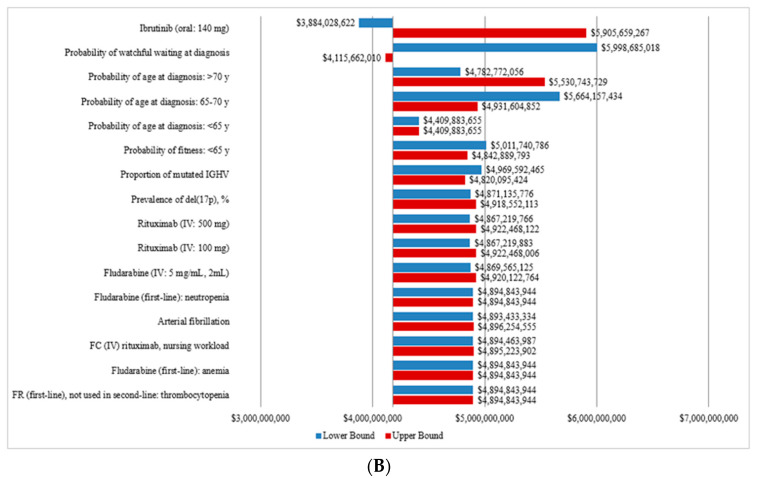
Sensitivity analysis of the total annual costs ^a^ of CLL for the CIT and the OTT scenarios. (**A**) Tornado diagram for the one-way sensitivity analysis of the total annual cost for the CIT scenario. (**B**) Tornado diagram for the one-way sensitivity analysis of the total annual cost for OTT scenario. CIT, chemoimmunotherapy; CLL, chronic lymphocytic leukemia; del(17p), deletion at chromosome 17p targeting the TP53 gene; FC, fludarabine, cyclophosphamide; FR, fludarabine and rituximab; IGHV, immunoglobulin heavy-chain variable; IV, intravenous; OTT, oral targeted therapy. ^a^ All costs are shown in 2019 Canadian dollars.

The OWSA showed that the cost of CLL management was most sensitive to the probability of age at diagnosis, the probability of WW, the treatment cost of rituximab and fludarabine, the probability of fitness for patients less than 65 years old as well as the management costs of certain AEs. The tornado diagram presenting the total costs of CLL management from 2011 to 2025 for the CIT and OTT scenarios are presented in Figure 4A,B, respectively.

## 4. Discussion

This study is the first to provide an analysis of the changing economic burden related to CLL management over time in Canada. Although OTT enhances survival for patients with CLL, it is nonetheless associated with an important economic burden due to the projected increase in costs from 2011 to 2025. This difference between scenarios is mainly due to the increased drug costs related to OTT. This study also projected a large increase in the number of people living with CLL over time, partially because of the increased incidence of CLL, but mostly because of the improved survival in the OTT scenario. Overall, this study estimates that the annual cost of CLL will increase to $957.5 million by 2025, a 15.7-fold increase from the annual cost in 2011. These findings directly show the economic impact on the healthcare system following the introduction of new OTT therapies. Altogether, our study highlights the impact of the rising CLL prevalence and the economic burden that would translate into enormous costs with the arrival of OTT onto the Canadian market.

A study published by Oliviera et al. in 2018 estimated the economic burden of cancer care in Canada [29]. In this analysis, it was observed that the cost of cancer care rose steadily over time, from $2.9 billion in 2005 to $7.5 billion in 2012. Therefore, according to these projections, the rising trend in the cost of CLL will most probably outpace that of other cancers. However, it is possible that advances in the research of other cancers will also increase their costs and overall economic burdens. Such increases could eventually become a problem for the entire budget of the healthcare system, including private payers and public reimbursements. 

Our findings are in line with a similar study conducted in the United States [12]. Chen et al. evaluated the cost-effectiveness of OTT from a population level and projected a 55% increase in the CLL population, from 128,000 in 2011 to 199,000 in 2025, and a 590% annual cost increase, from $0.74 billion to $5.13 billion, respectively. Prevalence and costs were estimated to increase over time, as was estimated in our Canadian study. However, percentage increases over time for prevalence and costs were found to be much higher in Canada, possibly because of differences in incidence rates or in drug costs between countries. 

Similarly, our study results were also in line with those published by Shanafelt et al. [30]. The purpose of this study was to assess the economic impact of the approval of ibrutinib and idelalisib in the treatment of CLL at the societal level, including an analysis on the out-of-pocket costs under Medicare Part D. This study projected the 10-year cost per treated patient to increase from $157,446 to $566,002, following the approval OTT in the United States. Our study confirms similar findings, with an estimated average increase of $43,309 per patient per year (approximately $433,090 over 10-years) in a Canadian setting [30]. Interestingly, Shanafelt et al. also assessed the out-of-pockets costs, which increased from $9,426 to $255,051, when considering the inclusion of OTT in first-line therapy. This study supports our results and further confirms the financial impact brought upon by continuous OTT, to the healthcare system as well as patients.

Our model has several limitations. First, although the model offers the possibility of selecting various treatment sequences, the results capture only one treatment regimen at a time. However, when selecting the various treatments options, the total costs varied within the same cost range. In addition, our analyses considered continuous OTT for all patient populations for which clinical studies demonstrated greater clinical value of continuous OTT compared to CIT. If OTT were limited only to subpopulations receiving the most benefits from this treatment, this would reduce the financial impact assessed within our study. Furthermore, not all patients are treated with the same treatment pattern and some treatments may deviate from the standard of care; nevertheless, four key opinion leaders across Canada participated in the development of this project, allowing the best possible treatment options to be selected and captured. Therefore, we strongly believe that our economic model sufficiently represents reality to the extent that it is representative of the true Canadian economic burden. Furthermore, we also considered constant drug prices over time and, although this is a limitation to our analysis, drug prices were included in sensitivity analyses and results remained consistent.

It is important to note that the purpose of the study was not to assess the cost-effectiveness, but rather the economic burden associated with the introduction of continuous OTT, in Canada. To assess the cost-effectiveness of continuous OTT, further cost-effectiveness analyses should be performed.

## 5. Conclusions

In conclusion, this study highlights the predicted increase in both the prevalence and costs associated with CLL in Canada. These will be substantially greater with the introduction of continuous OTT as a treatment option in addition to the previous standard of care, namely CIT regimens. The projected vast increase in costs will have an important impact on the healthcare system, particularly regarding the budget allocation. Changes in pricing or clinical strategies, such as the implementation of a fixed OTT treatment duration or discontinuation and retreatment based on depth of response, may help alleviate this financial burden.

## Figures and Tables

**Table 1:** Model and Cost ^a^ Parameters.

Parameters	Model	Reference
Probabilities, % (unless otherwise stated) Probability of WW at diagnosis	85.0	Assumption, Chen [12]
Median time to first treatment (years)	4.8	Parikh [2]
Transition probability from WW to first treatment by cycle	1.65	Model calibration
Proportion of patients in IV therapy	100.0	When both formulas available
Prevalence of del(17p)	7.0	Hallek [5]
Proportion of mutated IGHV	40.0	Confirmed by KOL
Proportion of non-mutated IGHV	60.0	Confirmed by KOL
Mean age at diagnosis	71	LLSC [1]
Probability of age at diagnosis		
Age <65 years old	33.5	Statistics Canada [9]
Age 65–70 years old ^b^	14.3	Statistics Canada [9]
>70 years old	52.3	Statistics Canada [9]
Probability of fitness		
Age <65 years old	90.0	Confirmed by KOL
Age 65–70 years old	50.0	Confirmed by KOL
>70 years old ^b^	0.0	Confirmed by KOL
Probability of discontinuing OTT for each 4-week cycle		
First-line treatment	0.7	Burger [13]
Relapsed patients	1.4	Burger [13]
Costs, $		
Laboratory monitoring costs		
Electrolyte panel	7.32	Code L226, 204, 053,165,194,061 [14]
Renal panel	2.56	Code L251,067 [14]
Liver function tests	10.39	Code L223, 222, 191,029,030, 031, 005, 208 [14]
CBC panel	3.98	Code L393 [14]
Coagulation parameters	6.20	Code LG031 [14]
Serology	10.25	CodeL319 [14]
Chemotherapy infusion, administration and management	105.15	Schedule of benefits. Code G359. [15]
Professional fees		
Consultation, hematology	157.00	Schedule of benefits. Code A615. [15]
Partial assessment hematology	38.05	Schedule of benefits. Code A618. [15]
Nurse average wage ($/min)	0.61	Job Bank Canada (NOC 3012). [16]
Pharmacist average wage ($/min)	0.89	Job Bank Canada (NOC 3131). [16]
Adverse events		
Anemia	4853	OCCI code D649. [17]
Neutropenia	7445	OCCI code D70. [17]
Thrombocytopenia	7572	OCCI code D69.6. [17]
Infection (viral\bacterial unspecified)	5802	OCCI code A49.9\B34.9. [17]
Arterial fibrillation	6546	OCCI code I48.90. [17]

CBC, complete blood count; del(17p), deletion at chromosome 17p targeting TP53 gene; IGHV, immunoglobulin heavy chain variable; IV, intravenous; KOL, key opinion Leader; LLSC, Leukemia and Lymphoma Society of Canada; OCCI, Ontario Care Costing Initiative; OTT, oral targeted therapy; WW, watchful waiting. ^a^ All costs are shown in 2019 Canadian dollars ^b^ Assumed that all patients older than 70 years old are unfit.

**Table 2:** Summary of Treatment-Related Parameters.

Treatment	PFS and OS	Adverse Events (%)	Drug Cost ^a^ ($/cycle)	Reference
**First-line setting**				
Clb	Median PFS, 18.0 months	Anemia, 27 Neutropenia, 12 Thrombocytopenia, 20 Infection, 4	Cycle 1: 249 Cycles 2–6: 166	Eichhorst [18]
F	≥65-years; Median PFS, 19.0 months	Anemia,15 Neutropenia, 12 Thrombocytopenia, 15 Infection, 80	Cycle 1-6: 1089	Eichhorst [18]
FR	Median PFS, 42.0 months	Anemia, 40 Neutropenia, 76 Thrombocytopenia, 20 Infection, 20	Cycle 1: 4454 Cycles 2–6: 5575	Woyach [19]
FCR	<65-years, 3-year PFS, 64%	Anemia, 6	Cycle 1: 4098	Hallek [5]
	≥65-years, 3-year PFS, 68%	Neutropenia, 30	Cycles 2–6: 5220	
	IGHV mutated, 3-year PFS, 80%	Thrombocytopenia, 9		
	IGHV unmutated, 3-year PFS, 55%	Infection, 24		
GClb	Median PFS, 26.7 months	Anemia, 4 Neutropenia, 33 Thrombocytopenia, 10 Infection, 12	Cycle 1: 16,493 Cycles 2–6: 5537	Goede [4]
Clb+R	Median PFS, 16.3 months	Anemia, 4 Neutropenia, 28 Thrombocytopenia, 3 Infection, 14	Cycle 1: 3,488 Cycles 2–6: 3365	Goede [4]
BR	≥65-years, Median PFS, 34.0 months	Anemia, 31 Neutropenia, 31 Thrombocytopenia, 35 Infection, 12	Cycle 1: 5,491 Cycles 2–6: 6612	Fischer [20]
Ibrutinib	6-month PFS, 90%	Anemia, 6	7615/cycle	Burger [13]
	Del(17p), 24-month PFS, 91%	Neutropenia, 10 Thrombocytopenia, 2 Infection, 6 Arterial fibrillation, 6		
**Relapse setting**				
F	Median PFS, 14.8 months	Anemia, 80	Cycles 1–6: 1089	Niederle [21]
	Median OS, 41.0 months	Neutropenia, 17 Thrombocytopenia, 60 Infection, 15		
FCR	Median PFS, 28.0 months	Anemia, 24	Cycle 1: 4098	Wierda [22]
	Median OS, 42.0 months	Neutropenia, 81 Thrombocytopenia, 34 Infection, 16	Cycles 2–6: 5220	
B	Median PFS, 20.1 months	Anemia, 4	Cycles 1–6: 2363	Niederle [21]
	Median OS, 43.8 months	Neutropenia, 20 Thrombocytopenia, 7 Infection, 13		
BR	24-months PFS, 41%	Anemia, 14	Cycle 1: 5491	Seymour [23]
	Median OS, 33.9 months	Neutropenia, 39 Thrombocytopenia, 10 Infection, 22	Cycles 2–6: 6612	Fischer [24]
Ibrutinib	30-month PFS, 69%	Anemia, 0	7615/cycle	Byrd [25]
	Del(17p), 30-month PFS, 48%	Neutropenia, 18		
	30 months OS, 79%	Thrombocytopenia, 10		
	Del(17p), 30 months OS, 65%	Infection, 51 Arterial fibrillation, 6		
Idelalisib+R	24-week PFS, 93%	Anemia, 5	Cycle 1: 10,838	Furman [26]
	1-year OS, 92%	Neutropenia, 34 Thrombocytopenia, 10 Infection, 0	Cycle 2: 11,959 Cycles 3–6: 7473 Cycles 7-progression: 2987	
Venetoclax	12-month PFS, 86%	Anemia, 11	Cycle 1: 1761	Seymour [23]
	Del(17p), 12-month PFS, 82%	Neutropenia, 58	Cycles 2–	
	24-month OS, 92%	Thrombocytopenia, 6	progression:	
	Del(17p), 12-month OS, 87%	Infection, 18	7615	Stilgenbauer [27]

B, bendamustine; BR, bendamustine and rituximab; Clb, chlorambucil; Clb+R, chlorambucil and rituximab; del(17p), deletion at chromosome 17p targeting the TP53 gene; F, fludarabine; FCR, fludarabine, cyclophosphamide and rituximab; FR, fludarabine and rituximab; GClb, obinutuzumab and chlorambucil; IGHV, immunoglobulin heavy-chain variable; OS, overall survival; PFS, progression-free survival; R, rituximab. ^a^ All Costs are shown in 2019 Canadian dollars.
